# Association of thrombocytosis with COPD morbidity: the SPIROMICS and COPDGene cohorts

**DOI:** 10.1186/s12931-018-0717-z

**Published:** 2018-01-26

**Authors:** Ashraf Fawzy, Nirupama Putcha, Laura M. Paulin, Carrie P. Aaron, Wassim W. Labaki, MeiLan K. Han, Robert A. Wise, Richard E. Kanner, Russell P. Bowler, R. Graham Barr, Nadia N. Hansel, for the SPIROMICS and COPDGene Investigators, Neil E. Alexis, Wayne H. Anderson, Igor Barjaktarevic, R. Graham Barr, Eugene R. Bleecker, Richard C. Boucher, Russell P. Bowler, Elizabeth E. Carretta, Stephanie A. Christenson, Alejandro P. Comellas, Christopher B. Cooper, David J. Couper, Gerard J. Criner, Ronald G. Crystal, Jeffrey L. Curtis, Claire M. Doerschuk, Mark T. Dransfield, Christine M. Freeman, Mei Lan K. Han, Nadia N. Hansel, Annette T. Hastie, Eric A. Hoffman, Robert J. Kaner, Richard E. Kanner, Eric C. Kleerup, Jerry A. Krishnan, Lisa M. LaVange, Stephen C. Lazarus, Fernando J. Martinez, Deborah A. Meyers, Wendy C. Moore, John D. Newell, Laura Paulin, Stephen Peters, Cheryl Pirozzi, Elizabeth C. Oelsner, Wanda K. O’Neal, Victor E. Ortega, Robert Paine, Nirupama Putcha, Sanjeev Raman, Stephen I. Rennard, Donald P. Tashkin, J. Michael Wells, Robert A. Wise, Prescott G. Woodruff

**Affiliations:** 1 Division of Pulmonary and Critical Care Medicine, Johns Hopkins University School of Medicine, 1830 E Monument St. 5th Floor, Baltimore, MD 21287 USA; 2 Department of Medicine, Columbia University College of Physicians and Surgeons, New York, NY USA; 3 Division of Pulmonary and Critical Care Medicine, University of Michigan, Ann Arbor, MI USA; 4 Division of Respiratory, Critical Care and Occupational Medicine, University of Utah Health Sciences Center, Salt Lake City, UT USA; 5 Department of Medicine, National Jewish Medical and Research Center, Denver, CO USA

**Keywords:** Exacerbations, Dyspnea, Quality of life, Platelet count

## Abstract

**Background:**

Thrombocytosis has been associated with COPD prevalence and increased all-cause mortality in patients with acute exacerbation of COPD (AECOPD); but whether it is associated with morbidity in stable COPD is unknown. This study aims to determine the association of thrombocytosis with COPD morbidity including reported AECOPD, respiratory symptoms and exercise capacity.

**Methods:**

Participants with COPD were included from two multi-center observational studies (SPIROMICS and COPDGene). Cross-sectional associations of thrombocytosis (platelet count ≥350 × 10^9^/L) with AECOPD during prior year (none vs. any), exertional dyspnea (modified Medical Research Council (mMRC) score ≥ 2), COPD Assessment Test (CAT) score ≥ 10, six-minute-walk distance (6MWD), and St. George Respiratory questionnaire (SGRQ) were modeled using multivariable logistic or linear regression. A pooled effect estimate for thrombocytosis was produced using meta-analysis of data from both studies.

**Results:**

Thrombocytosis was present in 124/1820 (6.8%) SPIROMICS participants and 111/2185 (5.1%) COPDGene participants. In meta-analysis thrombocytosis was associated with any AECOPD (adjusted odds ratio [aOR] 1.5; 95% confidence interval [95% CI]: 1.1–2.0), severe AECOPD (aOR 1.5; 95% CI: 1.1–2.2), dyspnea (mMRC ≥ 2 aOR 1.4; 95% CI: 1.0–1.9), respiratory symptoms (CAT ≥ 10 aOR 1.6; 95% CI: 1.1–2.4), and higher SGRQ score (β 2.7; 95% CI: 0.5, 5). Thrombocytosis was also associated with classification into Global Initiative for Chronic Obstructive Lung Disease (GOLD) group D (aOR 1.7 95% CI: 1.2–2.4).

**Conclusions:**

Thrombocytosis was associated with higher likelihood of prior exacerbation and worse symptoms. Platelet count, a commonly measured clinical assay, may be a biomarker for moderate-severe COPD symptoms, guide disease classification and intensity of treatment. Future longitudinal studies investigating the role of platelets in COPD progression may be warranted.

**Trial registration:**

ClinicalTrials.gov: NCT01969344 (SPIROMICS) and NCT00608764 (COPDGene).

## Background

Chronic obstructive pulmonary disease (COPD) is the third leading cause of death in the United States with multiple systemic manifestations and a disease trajectory affected by comorbid conditions, including cardiovascular (CV) disease [1, 2]. Platelets, long implicated in CV disease [3], are multifunctional and play a role in many pathophysiologic processes beyond hemostasis [4]. The pathogenesis of sustained platelet count elevation (thrombocytosis) involves either a myeloproliferative disorder or occurs as a reactive process secondary to an underlying neoplasm, chronic infection, inflammation, or physiologic stress [5]. Reactive thrombocytosis is a consequence of inappropriately elevated levels of thrombopoietin, which is up-regulated by the inflammatory cytokine interlukin-6, and has been associated with increased soluble markers of platelet activation [5, 6]. Previous animal studies have documented the role of platelets and platelet activation in bronchoconstriction, bronchial reactivity, airway inflammation, and remodeling and have corroborated clinical studies suggesting a role of increased platelet activity in allergic and non-allergic asthmatics [7].

In COPD, thrombocytosis and markers of platelet activation have been associated with disease prevalence and, recently, thrombocytosis has been associated with increased all-cause mortality in patients with acute exacerbation of COPD (AECOPD) [8–12]. Notably, prior research suggests that the platelet-mortality association is independent of cardiovascular outcomes suggesting an alternate pathophysiologic mechanism [10]. However, the role of platelets in COPD morbidity has not been explored. This study investigates the association of thrombocytosis with AECOPD, exercise capacity and patient reported respiratory symptoms and quality of life. The main hypothesis of this study is that thrombocytosis is associated with worse COPD morbidity. The large, well-characterized cohorts of the Subpopulations and Intermediate Outcome Measures in COPD Study (SPIROMICS) and the Genetic Epidemiology of COPD study (COPDGene®) are ideal populations in which to test this hypothesis.

## Methods

Participants with COPD (post-bronchodilator ratio of forced expiratory volume in 1 s to forced vital capacity [FEV_1_/FVC] < 70%) were selected from the SPIROMICS and COPDGene studies, two multi-center observational cohorts with mutually exclusive recruitment. SPIROMICS enrolled current and former smokers (≥20 pack-years) and nonsmokers (≤1 pack-year) aged 40–80 years from twelve clinical sites in the US with the goal of identifying intermediate outcome measures that predict long-term clinical endpoints of morbidity [13]. COPDGene enrolled self-identified non-Hispanic whites or African-Americans aged 45–80 years from twenty-one clinical sites throughout the US with ≥10 pack-year smoking history with the goal of identifying genetic factors associated with COPD [14].

In SPIROMICS, cross-sectional analysis of baseline data on complete blood count (CBC), symptoms, and recall of events from the year prior to the baseline visit were ascertained. In COPDGene, CBC was collected at a five-year follow-up visit and thus outcome data collected at the 5-year visit (current as of September 2016) were analyzed cross-sectionally. Thrombocytosis was defined as platelet count of ≥350 × 10^9^/L, a definition previously used in the literature [15–17]. All CBCs were performed at certified laboratories at respective sites.

### Outcomes

The primary outcome of interest was patient-reported AECOPD during the previous 12 months. Any AECOPD included events treated with antibiotics or oral corticosteroids as well as severe AECOPD, defined as symptoms requiring an emergency department visit or hospitalization. Secondary outcomes of interest included dyspnea (modified Medical Research Council questionnaire, mMRC) [18], COPD health status (COPD Assessment Test™, CAT) [19], exercise capacity (6-min walk distance in meters, 6MWD) [20], respiratory-specific quality of life (St George’s Respiratory Questionnaire, SGRQ) [21], and COPD disease burden based on Global Initiative for Chronic Obstructive Lung Disease (GOLD) criteria for symptom categories A-D [22]. Classification into GOLD group D, indicative of the largest symptom burden and highest risk for future exacerbations, was defined as at least one severe exacerbation or ≥2 exacerbations during the prior year and mMRC score ≥ 2 or CAT score ≥ 10.

### Statistical analysis

Differences in participant demographics, respiratory function, comorbid conditions, and univariate outcomes by presence of thrombocytosis were assessed using chi-squared test or Fisher’s exact test for proportions and t-test for continuous variables. Cross-sectional analyses were performed using multivariable logistic regression to model the relationship of thrombocytosis (dichotomized at platelet count ≥350 × 10^9^/L) with dichotomous outcomes including at least one AECOPD within the prior year, at least one severe AECOPD within the prior year, mMRC score ≥ 2, CAT score ≥ 10 and classification into GOLD group D. Multivariable linear regression was used to model the relationship of thrombocytosis with 6MWD and SGRQ score. SPIROMICS and COPDGene were analyzed separately then combined by meta-analysis using inverse variance weighting to produce a pooled effect estimate.

Potential covariates were tested including age (continuous), gender, race (African American vs other), highest level of educational achievement (less than high school vs high school or above), percentile of post-bronchodilator FEV_1_ (continuous) [23], smoking status (current vs former smokers), ambulatory hypoxemia (oxygen saturation after 6-min walk ≤90%), inhaled corticosteroid use (ICS), body mass index (BMI; categorical: underweight <18.5 kg/m^2^ vs normal/overweight 18.5 - <30 kg/m^2^ vs obese ≥30 kg/m^2^), anemia (dichotomous using gender-specific cutoffs of hemoglobin), and CV comorbidities (hypertension, coronary artery disease, congestive heart failure, diabetes, and stroke) using a change in the effect estimate ≥10% between the crude and bivariate models of the association between thrombocytosis and each outcome as criteria for inclusion in models [24, 25]. Age, gender, education, FEV_1_, smoking status, ambulatory hypoxemia, ICS use, anemia, diabetes, congestive heart failure, and hypertension met criteria for at least one outcome in either study and were included as covariates in all models. Because there is no standard definition for reactive thrombocytosis, sensitivity analysis was performed dichotomizing platelet count at 400 × 10^9^/L to test the robustness of the analysis. In a subset of SPIROMICS participants who had biomarkers measured concurrently with platelet count, the relationship of C-reactive protein (CRP) with thrombocytosis was explored using the Kruskal-Wallis test.

All analyses were conducted using SAS 9.4 (Carey, NC) except for the meta-analysis which was performed using Stata version 13/SE (College Station, TX). SPIROMICS and COPDGene were approved by the institutional review boards at each center and all participants provided written informed consent (ClinicalTrials.gov: NCT01969344 (SPIROMICS) and NCT00608764 (COPDGene)).

## Results

**Fig. 1:**
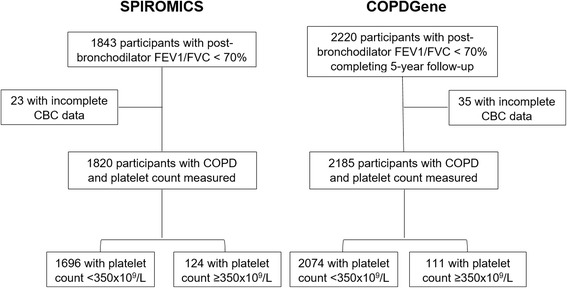
Consort diagram detailing the reason for exclusion of participants and ultimate classification by platelet count

In SPIROMICS, Thrombocytosis was detected in 124 (6.8%) of 1820 SPIROMICS participants and 111 (5.1%) of 2185 COPDGene participants with COPD (Fig. 1). Severity of lung disease, smoking status, supplemental oxygen use, and BMI were similarly distributed between COPDGene and SPIROMICS, although COPDGene participants were slightly older with higher prevalence of some comorbidities including anemia, hypertension, congestive heart failure, and diabetes. In both cohorts, compared to participants with platelet count <350 × 10^9^/L, participants with thrombocytosis were more likely to be female, have anemia and less likely to have coronary artery disease. In SPIROMICS, participants with thrombocytosis were also more likely to have less than a high school education and use supplemental oxygen while in COPDGene they were younger and more likely to be current smokers. Each of these trends was present but non-statistically significant in the other cohort (Table 1).

**Table 1:** Baseline characteristics and outcomes of SPIROMICS and COPDGene participants by platelet count

	SPIROMICS			COPDGene		
Characteristics [n (%) or mean ± standard deviation]	Platelet Count <350 × 10^9^/L (*n* = 1696)	Platelet Count ≥350 × 10^9^/L (*n* = 124)	*p*-value	Platelet Count <350 × 10^9^/L (*n* = 2074)	Platelet Count ≥350 × 10^9^/L (*n* = 111)	*p*-value
Age	65.2 ± 8	64.9 ± 7.6	0.7	68.3 ± 8.3	66.1 ± 8.6	**0.01**
Female	705 (41.6)	72 (58.1)	**0.0003**	894 (43.1)	72 (64.9)	**<0.0001**
Race: Black	254 (15.1)	21 (16.9)	0.6	456 (22)	27 (24.3)	0.6
Less than high school education	647 (38.3)	60 (48.8)	**0.02**	225 (10.9)	18 (16.2)	0.08
FEV1% predicted (post-bronchodilator)	61.3 ± 23.1	57.3 ± 21.5	0.07	60.5 ± 22.7	57 ± 22.6	0.1
GOLD I (FEV1% predicted ≥ 80%)	380 (22.4)	20 (16.1)	0.2	432 (20.8)	18 (16.2)	0.2
GOLD II (FEV1% predicted 50–79%)	753 (44.4)	55 (44.4)		933 (45)	50 (45.1)	
GOLD III (FEV1% predicted 30–49%)	389 (22.9)	38 (30.7)		508 (24.5)	26 (23.4)	
GOLD IV (FEV1% predicted < 30%)	174 (10.3)	11 (8.9)		201 (9.7)	17 (15.3)	
Current smoker	569 (34.2)	47 (38.5)	0.3	679 (32.8)	49 (44.1)	**0.01**
Oxygen saturation ≤ 90% after 6-min walk	541 (33.7)	43 (38.4)	0.3	653 (32.5)	33 (30.8)	0.7
Supplemental home oxygen	391 (23.2)	38 (30.1)	**0.05**	512 (24.7)	30 (27)	0.6
Anemia: Hemoglobin (g/dL) <13 (men) or <12 (women)	140 (8.3)	20 (16.1)	**0.01**	262 (12.6)	35 (31.5)	**<0.0001**
At least 2 cardiovascular comorbidities	303 (17.9)	21 (16.9)	0.8	484 (23.3)	20 (18)	0.2
Hypertension	853 (50.8)	62 (50.8)	1	1122 (54.2)	64 (57.7)	0.5
Coronary arterial disease	181 (10.8)	5 (4.1)	**0.02**	246 (11.9)	4 (3.6)	**0.008**
Congestive heart failure	53 (3.2)	2 (1.6)	0.6	111 (5.4)	8 (7.2)	0.4
Diabetes	226 (13.5)	22 (18)	0.2	353 (17)	13 (11.7)	0.1
Stroke	73 (4.4)	6 (4.9)	0.8	82 (4)	3 (2.7)	0.8
Body Mass Index (BMI, kg/m^2^)	27.4 ± 5.3	27.2 ± 6.1	0.8	28 ± 6.1	27.2 ± 6.3	0.2
Underweight (BMI < 18.5 kg/m^2^)	62 (3.7)	9 (7.3)	0.1	56 (2.7)	7 (6.3)	0.06
Normal/Overweight (BMI 18.5–30 kg/m^2^)	1108 (65.3)	75 (60.5)		1355 (65.3)	74 (66.7)	
Obese (BMI ≥ 30 kg/m^2^)	526 (31)	40 (32.3)		663 (32)	30 (27)	
Inhaled corticosteroid use	780 (46.5)	66 (53.7)	0.1	886 (43.1)	58 (53.2)	**0.03**

*GOLD* Global Initiative for Chronic Obstructive Lung Disease. Boldface indicates statistical significance at *p* < 0.05. Platelet count was measured at baseline in SPIROMICS and at 5-year follow-up in COPDGene

In unadjusted analysis in both cohorts, participants with thrombocytosis were significantly more likely to report an AECOPD (SPIROMICS: 43.6% vs. 30.7%, *p* = 0.003; COPDGene: 37.8% vs. 27.5%, *p* = 0.02) or a severe AECOPD (SPIROMICS: 24.2% vs. 15.5%, *p* = 0.01; COPDGene: 23.4% vs. 14.6%, p = 0.01) in the past year compared to the group with lower platelet count. Those with thrombocytosis were also significantly more likely to have worse dyspnea (mMRC score ≥ 2), functional status, quality of life (CAT score ≥ 10 and higher St. George Respiratory Questionnaire score), and more likely to be classified as GOLD group D compared to the group with lower platelet count (Table 2). Six-minute walk distance was reduced among participants with thrombocytosis in both cohorts but was statistically significant only in SPIROMICS.

**Table 2:** Univariate associations of thrombocytosis (platelet count ≥350 × 10^9^/L) with COPD morbidity

Outcomes [% or mean ± standard deviation]	SPIROMICS			COPDGene		
	Platelet Count <350 × 10^9^/L	Platelet Count ≥350 × 10^9^/L	*p*-value	Platelet Count <350 × 10^9^/L	Platelet Count ≥350 × 10^9^/L	*p*-value
Any Acute Exacerbation of COPD during the prior year	30.7%	43.6%	0.003*	27.5%	37.8%	0.02*
Severe Acute Exacerbation of COPD during the prior year	15.5%	24.2%	0.01*	14.6%	23.4%	0.01*
Modified Medical Research Council (mMRC) Questionnaire score ≥ 2	31.4%	47.5%	0.0002*	52.9%	64%	0.02*
COPD Assessment Test (CAT) score ≥ 10	72.4%	86.2%	0.001*	66.4%	77.5%	0.02*
GOLD symptom group D	20%	33%	0.0009*	19.4%	33.3%	0.0004*
6-min walk distance in meters	395 ± 128	352 ± 134	0.007^a^	371 ± 124	358 ± 118	0.3^a^
St. George Respiratory Questionnaire score	38 ± 20	44 ± 19	0.0002^a^	32 ± 22	38 ± 24	0.005^a^

*GOLD* Global Initiative for Chronic Obstructive Lung Disease. *chi-squared test; ^a^t-test

**Fig. 2:**
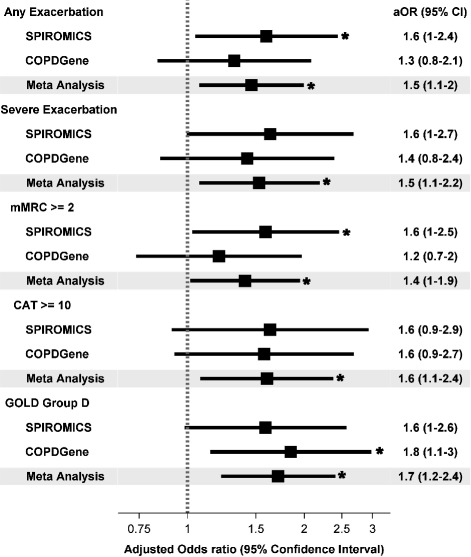
Association of thrombocytosis (platelet count ≥350 × 10^9^/L) with COPD morbidity (dichotomous outcomes). aOR: adjusted odds ratio; mMRC: modified medical research council questionnaire; CAT: COPD assessment test; GOLD: Global Initiative for Chronic Obstructive Lung Disease. Statistical significance (*p* < 0.05) denoted by asterisk

**Fig. 3:**
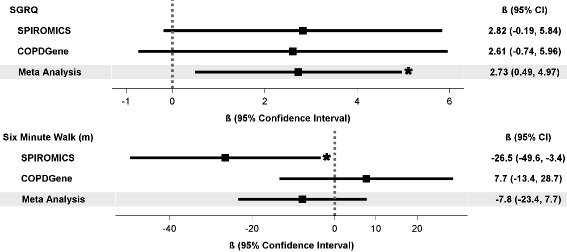
Association of thrombocytosis (platelet count ≥350 × 10^9^/L) with COPD morbidity (continuous outcomes). SGRQ: St. George Respiratory Questionnaire. Statistical significance (*p* < 0.05) denoted by asterisk

In adjusted analysis, compared with platelet count <350 × 10^9^/L, participants with thrombocytosis were 1.6 times more likely to report any AECOPD (95% confidence interval [CI]: 1.0–2.4) and severe AECOPD (95% CI: 1.0–2.7) in SPIROMICS, while results lost statistical significance in COPDGene for any AECOPD (adjusted odds ratio [aOR] 1.3; 95% CI: 0.8–2.1) and severe AECOPD (aOR 1.4; 95% CI: 0.9–2.4). Thrombocytosis was significantly associated with GOLD group D classification in COPDGene and with borderline significance in participants from SPIROMICS. Thrombocytosis was also significantly associated with mMRC ≥2 and reduced 6MWD in SPIROMICS. Among those with thrombocytosis, poorer health status and worse quality of life were present in both SPIROMICS and COPDGene, but differences between groups did not reach statistical significance (Figs 2 and 3).

In meta-analysis combining both cohorts, participants with thrombocytosis had a 1.5-fold increased odds of reporting any AECOPD (95% CI: 1.1–2.0) or severe AECOPD (95% CI: 1.1–2.2) during the preceding year, 1.4-fold increased odds of moderate-severe dyspnea (mMRC ≥2; 95% CI: 1.0–1.9), 1.6-fold increased odds of reporting worse COPD health status (CAT score ≥ 10; 95% CI: 1.1–2.4), and were more likely to be classified as GOLD group D (aOR 1.7; 95% CI: 1.2–2.4) (Fig. 2). Thrombocytosis was associated with worse respiratory-specific quality of life (2.7 points higher SGRQ score; 95% CI: 0.5, 5) but not 6MWD (Fig. 3).

Sensitivity analysis defining thrombocytosis as ≥400 × 10^9^/L (SPIROMICS *n* = 38, COPDGene *n* = 39) produced similar results for any and severe AECOPD, CAT ≥10, GOLD group D classification, and SGRQ, with results for mMRC ≥2 of similar magnitude to the main analysis but losing statistical significance (Table 3). Among SPIROMICS participants who had platelet count and CRP measured at baseline (*n* = 1041) participants with thrombocytosis had significantly higher median CRP (4.6 vs. 3.0, *p* = 0.01).

**Table 3:** Sensitivity analysis: the association of thrombocytosis with COPD morbidity using platelet count cutoff of 400 × 10^9^/L

	SPIROMICS	COPDGene	Meta-analysis
Dichotomous Outcomes [adjusted odds ratio (95% confidence interval)]			
Any Acute Exacerbation of COPD	1.7 (0.8–3.7)	1.8 (0.8–3.8)	**1.8 (1.0–3.0)**
Severe Acute Exacerbation of COPD	1.6 (0.7–3.8)	**2.7 (1.3–5.8)**	**2.2 (1.2–3.8)**
mMRC Score ≥ 2	1.5 (0.7–3.2)	1.5 (0.6–3.5)	1.5 (0.9–2.6)
CAT Score ≥ 10	1.9 (0.6–6.8)	**3.0 (1.0–9.2)**	**2.5 (1.1–5.7)**
GOLD Group D	2.3 (1.0–5.1)	**2.2 (1.0–4.8)**	**2.3 (1.3–3.9)**
Continuous Outcomes [β (95% confidence interval)]			
Six-minute walk distance (meters)	−12.5 (−53.8, 28.9)	−12.7 (−47.2, 21.7)	−12.6 (−39.1, 13.8)
St. George Respiratory Questionnaire score	**5.7 (0.4, 11.1)**	2.1 (−3.5, 7.7)	**4.0 (0.12, 7.8)**

Boldface indicates statistical significance at *p* < 0.05. *mMRC* Modified Medical Research Council; *CAT* COPD Assessment Test

## Discussion

This appears to be the first study assessing the association of thrombocytosis with exacerbations and patient-reported respiratory outcomes in a large, stable COPD cohort. In meta-analysis of two observational cohort studies (SPIROMICS and COPDGene), thrombocytosis was associated with increased odds of having AECOPD or severe AECOPD during the prior year, increased dyspnea, poorer health status, and worse respiratory-specific quality of life. Participants with thrombocytosis were also more likely to be classified in GOLD symptom category D. These findings, which persisted after adjustment for degree of airflow obstruction, CV disease, and other comorbid chronic diseases, suggest that platelet count, a routinely measured clinical assay, represents an objective measure associated with recent AECOPD and respiratory morbidity that may help guide treatment intensity in patients with stable COPD.

Elevated platelet levels have previously been shown to be more prevalent in patients with stable COPD [8], correlated with increasing airflow obstruction [26], all-cause mortality [10], and may be a surrogate for platelet activation [27]. In a study of 109 patients with stable COPD, platelet count was significantly higher than that of 51 healthy controls and did not differ by smoking status [8]. A strong correlation between platelet count and elevated levels of P-selectin, a glycoprotein expressed and secreted by activated platelets, has been reported [27], and a few studies have reported increased platelet activation in stable COPD measured directly as platelet-monocyte aggregates or as soluble markers of platelet activation [9, 28–30]. A recent study of patients hospitalized for AECOPD found that thrombocytosis was associated with increased risk of in-hospital and 1-year mortality independent of cardiovascular events and correlated with respiratory failure and exacerbation severity [10]. Among 452 individuals with stable COPD, mean platelet count increased with increasing severity of airflow obstruction [26]. Despite these few studies, there has been limited data to date evaluating whether platelet levels are associated with other measures of COPD morbidity.

Findings from this study show that thrombocytosis is linked to several respiratory outcomes in patients with COPD, including increased prevalence of exacerbations and worse dyspnea and respiratory-specific quality of life. Inclusion of CV comorbidities as a covariate in models did not eliminate or substantially change the association between platelet count and outcomes and thus is likely not a major mediator of the association. These findings further support the likelihood that the pathophysiologic mechanism underlying the association of platelets and COPD morbidity is independent of increased cardiac risk. In addition, some prior studies have reported higher platelet counts among smokers compared to non-smokers, however findings have been inconsistent [8, 31–33]. Approximately one-third of the study participants reported actively smoking and thrombocytosis was associated with smoking status only in COPDGene. However, inclusion of smoking status as a covariate in all models implies that the association of thrombocytosis with COPD morbidity does not simply reflect increased COPD morbidity among current smokers.

Several alternative mechanisms have been proposed for the role of platelets in COPD that could potentially inform the association with AECOPD. COPD is now recognized as a disease involving systemic inflammation [34], which increases further during AECOPD [35] with persistent elevation of inflammatory markers among non-responders, frequent, and recurrent exacerbators [36]. Platelets act in the inflammatory pathway by releasing pro-inflammatory mediators and activating other inflammatory cells [37]. Prior studies have shown a weak non-significant correlation between platelet count measured as a continuous variable and CRP in both stable [8] and exacerbated [10] COPD, however, in this study thrombocytosis was associated with significantly elevated CRP, as participants with thrombocytosis had significantly higher median CRP (4.6 vs. 3.0, *p* = 0.01), implying that systemic inflammation may play a role in the pathway between platelet elevation and COPD morbidity. It remains unclear whether AECOPD elicit a reactive thrombocytosis leading to inflammation through increased platelet activation and cytokine release or if systemic inflammation precedes reactive thrombocytosis and AECOPD.

Additionally, pulmonary endovascular abnormalities are prevalent in COPD and are present even in mild disease [38]. Studies have shown endothelial dysfunction, intimal thickening, as well as reduced and narrowed vasculature in COPD, leading to impaired relaxation and gas exchange and promotion of platelet rolling and aggregation [39–41]. Platelets also play a role in angiogenesis, maintaining vascular integrity, and release vasoactive substances [37]. Prior studies reported increased platelet count among hypoxic patients with airflow obstruction [42], possibly due to increased interaction and activation of platelets driven by compromised endothelium. The present study demonstrates that thrombocytosis was independently associated with AECOPD after controlling for ambulatory hypoxemia. While this may suggest that the association of platelets with morbidity is independent of endothelial dysfunction, hypoxemia is multifactorial and only one of several physiologic manifestations of endothelial abnormalities. More precise and direct measures of endothelial dysfunction are required to elucidate platelet-endothelial interactions in COPD.

There are limitations to this study. Given the cross-sectional nature of this investigation, it is not possible to determine temporality and it remains unclear whether thrombocytosis is a risk factor for or physiologic reaction to AECOPD or disease severity. Without a clear clinical cut-off for defining thrombocytosis, misclassification based upon the chosen value is possible, however, any misclassification would attenuate the associations described in this study toward the null. Furthermore, these findings were robust to a sensitivity analysis using a higher cutoff for defining thrombocytosis, even though analysis of mMRC score lost statistical significance owing to the low prevalence (approximately 2%) of platelet count greater than 400 × 10^9^/L. In a substudy of SPIROMICS participants (*n* = 98) undergoing repeat baseline evaluation 2–4 weeks from the original visit a change in exacerbation history recall of the previous year occurred in 30% and 13% of participants reporting total and severe exacerbations respectively, with repeatability of other measures ranging from fair (mMRC) to excellent (SGRQ) [43].

## Conclusions

Thrombocytosis was independently associated with worse COPD morbidity, including higher likelihood of prior exacerbation, worse patient reported outcomes, and GOLD group D classification. While the mechanism behind this association remains unclear and requires additional investigation, increased platelet activation perpetuating local and systemic inflammation may play a prominent role in COPD morbidity. These study results suggest that platelet count, a routinely measured clinical assay, may be an important biomarker associated with AECOPD and respiratory morbidity in patients with previously stable COPD that can aid in classification of disease severity and treatment intensity.

## Abbreviations

6MWD: Six-minute-walk distance
95% CI: 95% confidence interval
AECOPD: Acute exacerbation of chronic obstructive pulmonary disease
aOR: Adjusted odds ratio
CAT: COPD Assessment Test
COPD: Chronic obstructive pulmonary disease
COPDGene: Genetic Epidemiology of COPD study
CRP: C-reactive protein
FEV_1_: Forced expiratory volume in 1 s
GOLD: Global Initiative for Chronic Obstructive Lung Disease
mMRC: Modified Medical Research Council questionnaire
SGRQ: St. George Respiratory questionnaire
SPIROMICS: Subpopulations and Intermediate Outcome Measures in COPD Study
